# The Control of Focal Infections*Presented before the fiftieth anniversary meeting of the Dental Society of the State of New York. Saratoga Springs, N. Y., June 13-15, 1918. Reprinted from *Dental Cosmos* for November.

**Published:** 1918-12

**Authors:** C. H. Mayo

**Affiliations:** Rochester, Minn.


### THE CONTROL OF FOCAL INFECTIONS *

BY C. H. MAYO, M.D., ROCHESTER, MINN.

All life, from the standpoint of health and disease, is cell life. The chemistry of the world's activities is developed through the functional activity and protoplasm of the cell. The first forms of life, unicellular organisms, are lawless in their activities, multiplying without limit as food and environment is secured, and the stronger destroying the weaker. The normal type of microbe lives on the weaker animal and on the plant type of life, completing its existence from lack of food or the resistance of the host. It then dries into spore form, to again spring into action under suitable conditions. The lawless existence in unicellular organisms in contrast to multicellular life does not occur naturally. When the multicellular organisms appeared, true death entered the world. Under necessary control of growth and function through community existence, they became the prey of the unicellular organism. Man should not complain of the action of these organisms, because through them occurs the evolution of the world, and the bad effects of certain germs under abnormal conditions may be far outweighed by the good they do.

If one considers the countless numbers of unicellular organisms, many so small as to require the highest power of the microscope to be seen, and many of whose existence we know and yet have failed to identify, it will be seen that, of these, few in proportion to the total number are destroying agents. The greater part of the disease germs are under the control of man's intelligence, if he has the power to enforce the preventive measures known

---

* Presented before the fiftieth anniversary meeting of the Dental Society of the State of New York, Saratoga Springs, N. Y., June 13-15, 1918. Reprinted from *Dental Cosmos* for November.

to the world today. It is through such measures, applied in earlier life, that during the last thirty years the life of man has been lengthened a number of years. The microbes causing disease in man eventually bring about a stage of his life in which sudden death occurs from affections of the heart, brain and kidneys, between the ages of fifty-two and sixty-two years, as we have in no way changed middle age or advanced many more into old age. Death which is not accidental is due to the effects of the action of microbes—a result that may be acute and sudden, or chronic and slow in its termination.

The contagious character of various diseases has been appreciated for untold ages, and it has been known that certain of them developed some change in the individual which rendered him immune to a second attack of the disease. The first disease for which a vaccine was developed was that of smallpox and while used in China and India long ago, it was first used in Europe in Belgrade, and was brought to the English-speaking people by the discoveries of Jenner.

A study of the blood in disease, as varying from its condition in health, and the action of its cells in developing anti-bodies, has been of wonderful value to mankind. Through this study, acute diseases that create an immunity are reduced in morbidity and mortality by increasing the resistance of the patient, as in tetany, typhoid, partyphoid, typhus, yellow fever, etc. The innumerable diseases that formerly decimated mankind have been almost driven from the earth.

It is because of this wider knowledge of medicine that it has been possible to continue the present war without the enormous armies being destroyed by diseases which would long since have brought the war to an unsatisfactory termination.

We find, then, that there is developed in the blood stream, in acute diseases and fevers, an immunizing agent.

On the other hand, with certain diseases there is, at some place in the body, a small focus of bacteria continually maintained, developing not an immunity, but an anaphylactic reaction of the constant supply of microbes or microbe toxin instead of elevating the resistance of the patient against the germ. Such persons are subject to recurring colds on the slightest provocation, recurring neuralgias, recurring myositis, muscular rheumatism so-called, lumbago, sore muscles of the back and neck, etc., and it has been enough in the past for the patient to say he is subject to such trouble and for the doctor to make local applications and allow time to complete the cycle of improvement until, from any cause, lowered body resistance again reinstates the liability to an attack—and any part once affected by a microbe becomes more liable to repeated attacks.

We have also protein poisoning. Many persons are unable to eat various grains or berries, milk, fish, etc., which cause them to develop asthma or chronic diseases of the respiratory tract, or of the mucous membrane or skin, shown by local swelling, diarrheas or eczema.

Although there are but few places in the body in which man quite regularly carries bacteria, they are always in the mouth, often in the tonsil and about the teeth in pyorrhea, alveolar abscesses, and buried crypts of tonsils. All tonsils that are capable of reacting to the infection and are of good size, three or four on the scale of four, are usually not the cause of chronic disease but of strictly local involvement, and when inflamed temporarily, develop systemic disturbances. The position is most difficult for many physicians who have but recently come to a knowledge of the danger of a focus in these instances, not realizing that the blood stream is the carrier of the infection. In such cases the localizing trouble in the sciatic nerve or in the joint did not begin there, but arose from the existence of bacteria in a minute pocket, and if that pocket is under tension the disease is essentially chronic. The physician examines the throat and says the tonsils are not inflamed, or are graded one or two in size, and can not be the source of the trouble. The dangerous tonsil is the one graded one or two, without any effects of local inflammation on its surface.

Diseased teeth are often local foci of infection, and the X-ray has been of inestimable value in determining the presence of alveolar abscesses, absorbed roots of teeth, or absorbed bone about the roots. The findings are striking, when positive, but many pockets do not show in an apparently good picture. The dangerous tooth is a crowned tooth, and if it is necessary, from the seriousness of chronic, recurring diseases which affect the heart, as a myocarditis, or the kidneys, or the joints or nerves, then small tonsils must be removed and teeth most carefully inspected, X-rayed, and, when diseased, extracted on the basis of symptoms, should they be of major importance. Endarteritis, overgrowth of bone about the joints, including the hip and spinal vertebræ, are also due to minor types of bacteria, which are probably in pockets not under tension. I have far less fear where nature loosely holds the bacteria; the dangerous ones are always under tension and in small areas, although we must now come to the acceptance of the fact that the blood of apparently healthy persons often contains microbes.

In our bodies, with almost no evidence of it, are living and growing the amœba, the syphilitic spirochete in almost every place, the hookworm, and other germs too numerous to mention, and often temporarily doing no more harm than trout in spring water. We have wandering leucocytes with almost the power of animals to leave the blood stream, forage for material dangerous to life, and return to the circulation; and in many of us, a little blood drawn, and time given for culture, will show some kind of microbe to be present. The stomach does not destroy all the bacteria taken into it; some may pass into the blood by the chyle duct, and probably more commonly enter the blood stream by way of the portal circulation but are destroyed in the liver. The germs in the mouth are carried on into the stomach, and in the great majority of persons there are numerous bacteria living in the gastric juice after all food has left the stomach. The dangerous varieties are those of the acid type, while those of alkaline nature are nuisances.

Of secondary importance to the microbe, from a biologic standpoint, is the chemistry of the fluids of local areas for their environment. This is similar to the result from seeds planted or blown upon different soils. They may be planted to no purpose on the wrong soil, and they may be blown everywhere to take growth to advantage in proper environment. Bacteria carried throughout the body by the circulation are able to take up local growth only when thus carried to a given area. This accounts for the specificity of bacteria in their location causing acute and self-limited diseases, or chronic recurring or relapsing diseases. The acidity, the oxygen tension, and the condition of the general health, or local injury, may all be factors. Some forms will only grow in a certain place, as poliomyelitis in the brain and spinal cord, others in the sheaths of nerves, the first causing acute conditions, self-limited, and the latter, recurring neuritis. Thus we have rheumatism, appendicitis, gall-bladder, inflammations and ulcers of the stomach, valvular diseases of the heart; in fact, nearly all of the local and general diseases of which we have knowledge are thus produced.

The factors of safety are largely within the control of man, in preventing diseases, and in the transference of immunizing resistant bodies, such as have been developed for the cure and prevention of diphtheria, typhoid fever, smallpox, poliomyelitis, and many other affections.

Diseases of middle life are increasing. They are microbic, of a chronic, recurring character and are carried into the blood stream from a few foci, the mouth being the source of greatest danger. A crowned tooth is not a crown of glory, and may cover a multitude of germs. Modern dentistry is relieving the world of much of its misery by watchful care of foci connected with the teeth, and the trend of modern medicine and dentistry is bringing their fields again closely together. Dentistry should be a department of medicine, as it is as closely associated with medicine as are the specialties of the eye, ear, nose and throat, etc.

DISCUSSION

Dr. M. L. Rhein: The question of the control of focal infections is unquestionably a very practical one, as far as it concerns dentistry. The question is, What are we, as dentists, to do under the circumstances? Dr. Mayo has divided the subject into two phases, one preventive and the other curative—a division that is undeniably the best that we could make. There is not a point in the splendid paper to which we have just listened that I can imagine exception being taken to. Of all the essayist has said, the most impressive thing to me was his reference to the subject of the prevention of focal infections. This is no new topic to dentists; it is almost as old as dentistry itself, and it is the one thing that we ought to give the greatest attention, in order that the coming generations shall be brought up in such a way that focal infections can not take place, which means that there shall be no devitalized pulps—because, when you analyze the subject, it rests there.

I was particularly impressed by the division the essayist made when he spoke of abscesses. Personally, I prefer to divide this subject, in treating of its symptomatology, into abscesses and what we were formerly accustomed to calling "blind" abscesses. The essayist divides them into those in which the bacteria are loosely held, as against those that are under tension, and he speaks most forcibly of the greater danger of those in which the bacteria are under tension.

The ordinary alveolar abscess is one where we have suppuration, where the toxins are germinated from the pus, and are not under tension. Nature has already started in to play the role of doctor to cure the patient. The pus finds an outlet in the mouth, and is swallowed, and to a certain extent this action is nullified by the gastric juices. The alveolar abscess is comparatively harmless as compared with the "blind" abscess. Dr. Mayo has noticed this clinically, and it is entirely in accord with all we have noted in the literature on the subject. In the "blind" abscess we have no suppuration. We have the colonization of bacteria, locked in about the roots at the ends of the teeth, and during mastication and the movement of the teeth we get the tension he speaks of against this habitat where the bacteria do not proceed to cause suppuration, because no inflammation sets up under these conditions; but they in turn produce the same toxins that are produced by the alveolar abscess, only in this case there is no outlet for this poison. It remains securely locked in this comparatively small space, and the safety of the individual is dependent entirely upon how immune he or she may be against these poisons. Nature provides for the defense of the individual by the cell life that he speaks of, and it is only when these defensive conditions are weakened—it may be by disease, it may be by over-exercise, or it may be through some local cause; but when this defensive power is lessened, then is the time these toxins are taken up by the circulation and carried to some special point, and their manifestation becomes a more or less serious or perhaps a critical one.

The prevention of this condition is the one thing we, as a profession, should give greater consideration to than we have done in the past, because it is the only positive thing we know today that will assure the ultimate preservation of the natural teeth. That means starting with the teeth before the child is born— starting with the education of the mother in regard to the character of the food that makes for proper teeth in the child. From the time the first teeth are erupted there comes into play the question of focal infection, and that involves the broad question of what may be termed the prophylaxis of the mouth. With this subject we are all familiar, and it is the one great means that dentists must in the future utilize beyond what we are doing at the present time, if we would do our share, as far as our responsibility is concerned, toward the welfare of the teeth of the future race of our country.

In regard to the curative question—and that is the difficult one that confronts us as a profession—the point I would start with is that the dentist is not educated to handle these cases properly when they first present themselves. The dentist has not been instructed in making a proper, careful diagnosis of a mouth where a focal infection is suspected, or where one may exist. Too frequently, when there is just one tooth in question that stands out with a focal infection, the dentist goes right to work at that tooth, and pays very little attention to the rest of the mouth. There may be a dozen other focal infections in the same mouth; that is a point that should be well understood. What does a diagnosis mean? It means, first, that the dentist should have a chart of the mouth that indicates absolutely every tooth in which a devitalized pulp is present, or suspected to be present, and to demonstrate to himself whether such a condition exists; there is nothing of more importance than this.

The next step is to make a diagnosis of the conditions, the symptomatology; whether it is a case in which a pyorrheal condition is combined with a focal condition, or whether perchance there may be a pericemental infection without a devitalized pulp. To make this examination properly is a matter that can not be done in a few minutes, and frequently not in a few days. It means the proper roentgenographing of the mouth. It means an understanding of the proper physical condition of the individual. A dentist who is handling a case of that kind should have at his command the urinalysis of such a patient, the blood examination of such a patient, and the opinion of the internist, if there be one in the case. All of those questions become important matters in the decision that he must come to, after he understands properly what the condition of each particular tooth may be. If, for instance, we have a patient in whom the teeth to all intents and purposes, physically, as they express themselves to our view, present the very best of appearances—as I have frequently seen them, where before the use of the X-ray we would never have dreamed of a focal infection—and yet that individual is suffering from marked toxemia, a very marked diabetic condition is present, or a nephritis in a marked degree that has alarmed the internist. That is no case, in my opinion, for prolonged dental treatment—however successfully we may be able to save those teeth, if the condition of the patient were not so serious.

In many of those cases it is important to know what the physical condition is—not as we, as dentists, look at it, but as the physician looks upon it; and it is for that reason that focal conditions of infection should be handled only co-operatively by the physician and the dentist. Often when we know from a careful examination of the roentgenogram of an infected tooth that we have the ability to put that tooth in a healthy condition and to keep it free from infection, we should realize that before that can be properly accomplished we may endanger the life of the patient! Under such circumstances, I do not think there is an individual present who, if he fully realized that fact, would think of attempting to save such a tooth. In other words, the value of the tooth becomes insignificant—much as we dentists may value it—when we compare it with the life of the individual.

Granting that fact, we now come to another stage of the question of the control of focal infection. We have before us, we will assume, an individual in whom the blood examination, urinalysis, and everything else is of such a nature that the internist says, "If you can save those teeth, go ahead and save them." It is now up to the dentist to determine, "Can I put the tooth, or the teeth, in such condition that these focal points will be not only free from infection, but will remain free from infection?" I contend that it is only partially doing our work if we simply treat those teeth so as to eradicate the infection and not be secure in the belief that they can not become reinfected. Today I believe that it is almost impossible for any man, after he has completed the eradication of focal infections and put the most valuable kind of work into the operation, and has made a root-filling that meets his satisfaction, to say immediately on the conclusion of the operation that that tooth is safe from infection. I do not believe he can give a safe opinion in regard to a tooth of that kind until from six to twelve months have elapsed. In other words, the only rational proof we have is that there shall be a regenerated alveolar structure where the bone was destroyed, and that it shall remain in that condition. If we fail to secure that result the operation is a failure. Today I do not believe in my own mind that a large number of teeth with devitalized pulps are fit to remain in the human jaw, although fifteen years ago I felt calmly secure in the belief that they could be placed in a healthy condition. I have reduced in my own mind the percentage of such teeth that are fit to remain in the mouth, and while I realize that this is an extremely discordant note to introduce to a body of dentists whose aim is the preservation of the human tooth for the value it has to humanity, yet I can only appeal to you, my brother dentists, in this way: That it is our duty to become big enough in the moral sense to outgrow the natural smallness of all specialists who see only the small field in which they are interested, but constantly to keep before our minds the thought that we, as physicians of the teeth, are just as certainly physicians of the body. We can not isolate a part from the whole, and when we imperil life we are no credit to ourselves. I do not think there is a man or woman in dentistry who values a human tooth more than I do, and I realize the wrongs I have done innocently in this respect and it is for that reason I say we must be careful not to retain in the mouth teeth that are a menace, or are liable to be a menace, to the life of an individual.

The argument is frequently made that as long as the loss of the alveolar structure is not progressive, as long as we can watch such a tooth by means of the roentgenogram, we should keep those teeth, and keep them under observation. Let us stop for a moment and think what it means to keep the teeth of our patients under roentgenographic observation. Is that a practical matter with the majority of our patients, to say the least? You know it is not. Who knows where that patient may be when that immunity to this toxemia may break down? Only three weeks ago I extracted two molars from the mouth of a woman around the age of sixty years, in which I filled the roots about twenty-five years ago. They were teeth filled with nodular pulp matter in such a manner that I failed to reach the foramina of those teeth. I knew it at the time. I spent hours in the treatment of those teeth. I have radiographed those teeth in the past ten years a dozen times, and never found anything but healthy alveolar structure there until about three months ago, when the patient, presenting herself, as she did on regular occasions, for prophylactic treatment, sent word in to me that she thought she would like to have those teeth radiographed again. To my astonishment, I found marked focal infections at the ends of the roots of both those molars. Those teeth were extracted within forty-eight hours. There is a case where the patient of her own accord entirely entered into the question; but how often are we able to find such a condition in this way? I bring up the case as a practical one, to show the fallacy of depending on healthy alveolar structure where we know the root-canal work is intrinsically defective, and what the possibilities in the case of such work may be in the future.

This is no question that can be settled within the few moments allotted to it for discussion, but I feel that if I leave this point with you, I have accomplished a great deal—namely, there is no such thing as safety in the control of focal infections unless the focal infection is eradicated and the end of the root is hermetically encapsulated, so that it is absolutely free from the possibility of microbic attack in the future.

Dr. D. W. McLean: A patient comes into our office, and we remove a tooth. The patient tells us when we see him again that he was laid up with a severe cold, or an attack of grip or rheumatism, following the extraction. What does that mean? It means a focus of infection. It means we have stirred up a hornet's nest in the alveolar tissue surrounding that tooth, that there has been a fresh invasion of the patient's system, and the reserves were called out; and while the reserves and the germs were fighting it out, the patient went to bed; he was sick.

There lies our tremendous responsibility. We are fond of criticizing the medical profession for the tendency some of its members have to go over our heads and condemn teeth. About a week ago a patient came into my office and said she wished a root extracted, because she was suffering from rheumatism. The physician had examined the mouth, and the root was the only thing he found there to cause the trouble. There was a great deal of pyorrhea, but the patient was quite indignant when I spoke of it, and said the physician had examined the mouth and said there was no pyorrhea! We all have had cases where the physician has sent the patient to the radiologist and has made his dental diagnosis as well as medical, and we recall the fact that that is practicing dentistry, and even a physician can not practice dentistry without violating the law of the state. But he is often justified. Suppose you were a physician treating a case of focal infection with secondary lesions. Suppose you had eliminated almost everything else, and you felt sure the trouble was in the mouth and the teeth, and you sent your patient to a dentist who for the past ten years had been suffering from mental ankylosis. What would you do? You would be justified in taking that patient away from that dentist and putting him in more competent hands. Very often, however, the same condition exists the other way around.

I have been treating and I hope to show you the radiographs of a patient with acute arthritis. We have eliminated every focus of infection in the mouth with the exception of one suspicious vital pulp. So far we have found nothing. That patient ought to be in the hands of a competent group of men working together. I have referred her to her physician—I did so early in the treatment of the case—and he said her rheumatism "came from the air." What should the dentist do in a case like that? The subject of focal infections lies out in No-man's Land between the two professions, *and neither one alone* can treat it thoroughly and to its full completion.

I was impressed with Dr. Rhein's statement as to the prevention of focal infection; and the body of men in this State Society today that is doing the biggest work toward saving people from these focal infections is your Oral Hygiene Committee. It boils down to a question of prevention; of examining the teeth frequently. We must spend our time filling pinhole cavities, instead of treating pulps—or capping them, which I believe to be equally bad.

